# Supplementary material for: A multi-tetracycline responsive induction system for gene expression in Bacillus subtilis
Source: Appl Environ Microbiol. 2026 Apr 23;92(5):e00210-26. doi: 10.1128/aem.00210-26 (PMC13188878; doi:10.1128/aem.00210-26)
Supplement: Supplemental material — Figures S1 to S14 and Tables S1 to S4. [file aem.00210-26-s0001.docx]

**Supplementary Information**

**A Multi-Tetracycline Responsive Induction System for Gene Expression in *Bacillus subtilis***

Keira Reich-Veillette^1^, Amrita E. Rhoads^1^, Elizabeth A. Libby^1*^

^1^Department of Bioengineering, Northeastern University, Boston MA 02115 USA

*corresponding author

**Supplementary Figures**

**
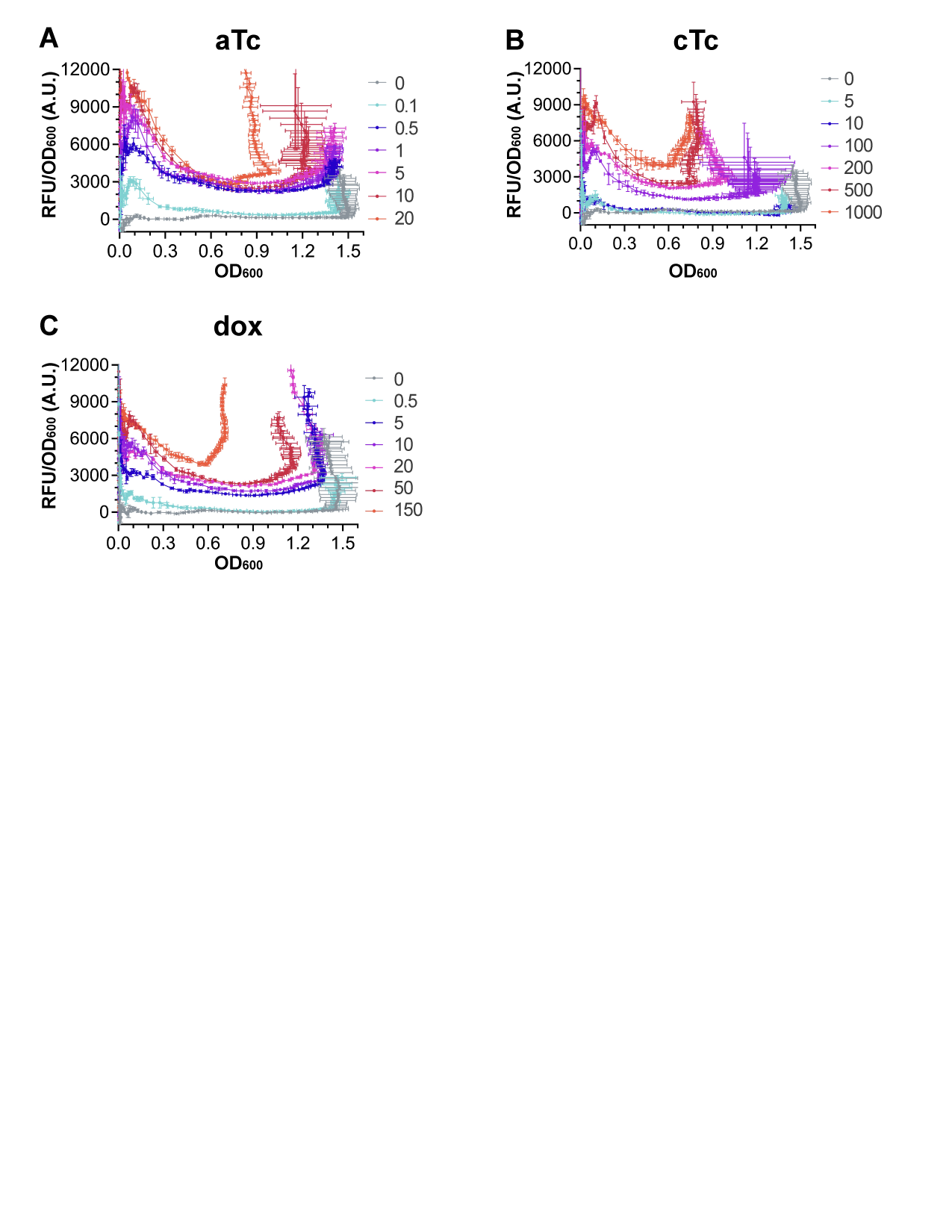
**

**Figure S1:** **Tetracycline induction throughout growth in minimal glucose media.** Representative mean mCherry fluorescence for (A) aTc, (B) cTc and (C) dox (ng/mL) in minimal glucose media during bacterial growth. Error bars represent standard deviation across triplicates within a single experiment.

**
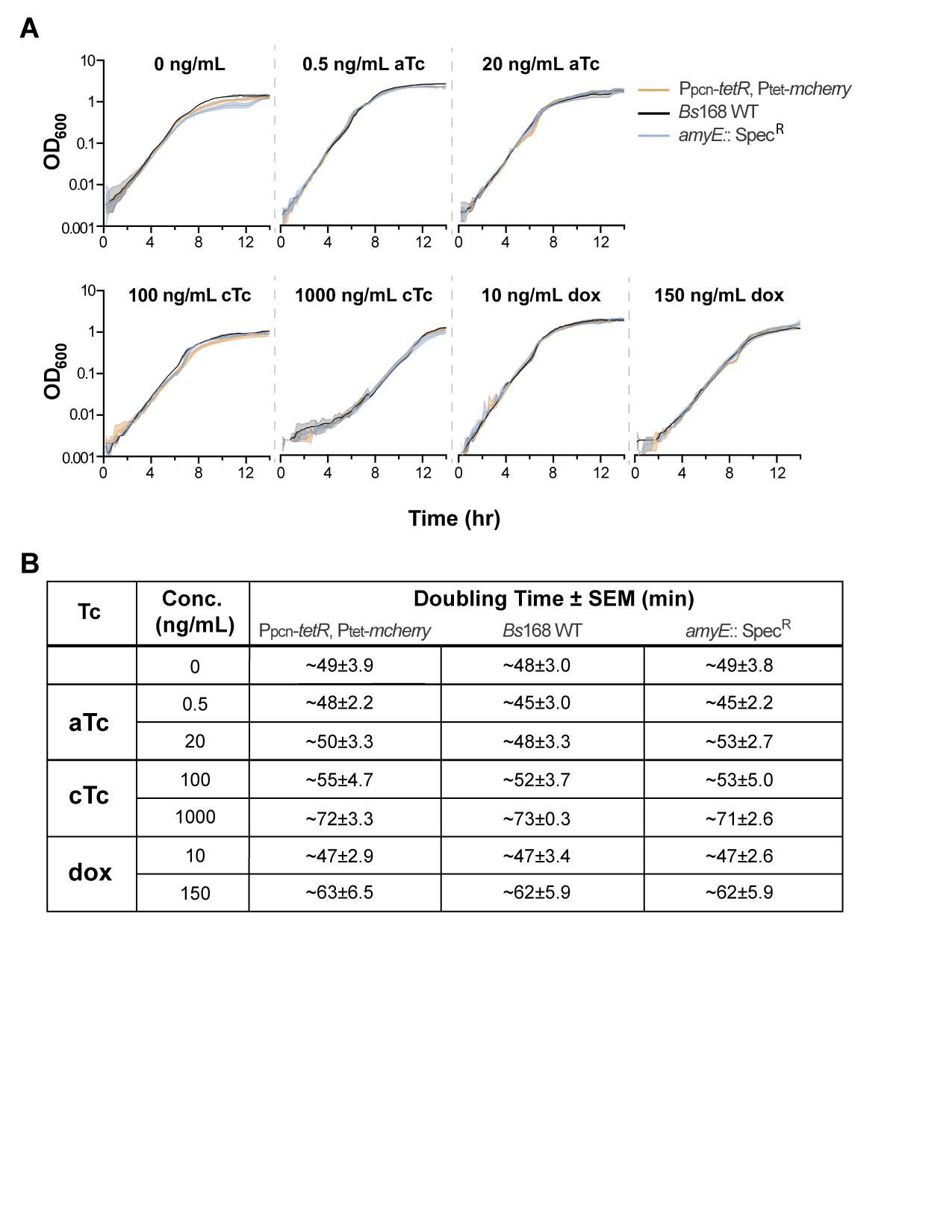
**

**Figure S2:** **Growth curve for strains harboring the inducible system in minimal media with tetracyclines.** (A) Representative growth curves for 0, ~EC50, and maximum inducer tested for each tetracycline in minimal media. Error bars represent standard deviation across triplicates within a single experiment. *Bs168* and integration of an empty vector at the same chromosomal locus are included as controls. (B) Corresponding mean doubling times and SEM in log phase (through OD_600_ ~0.15) across three biologically independent experiments.


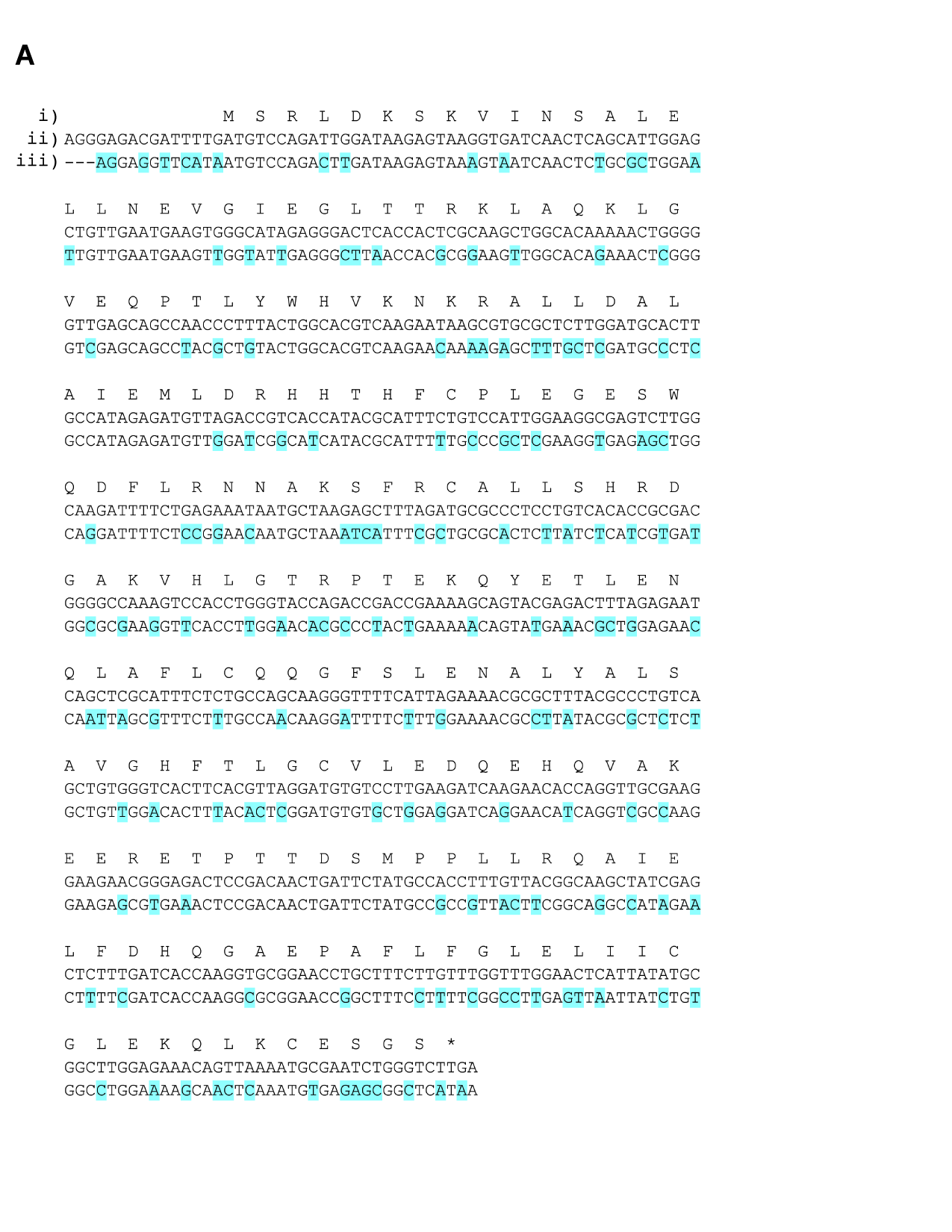


**
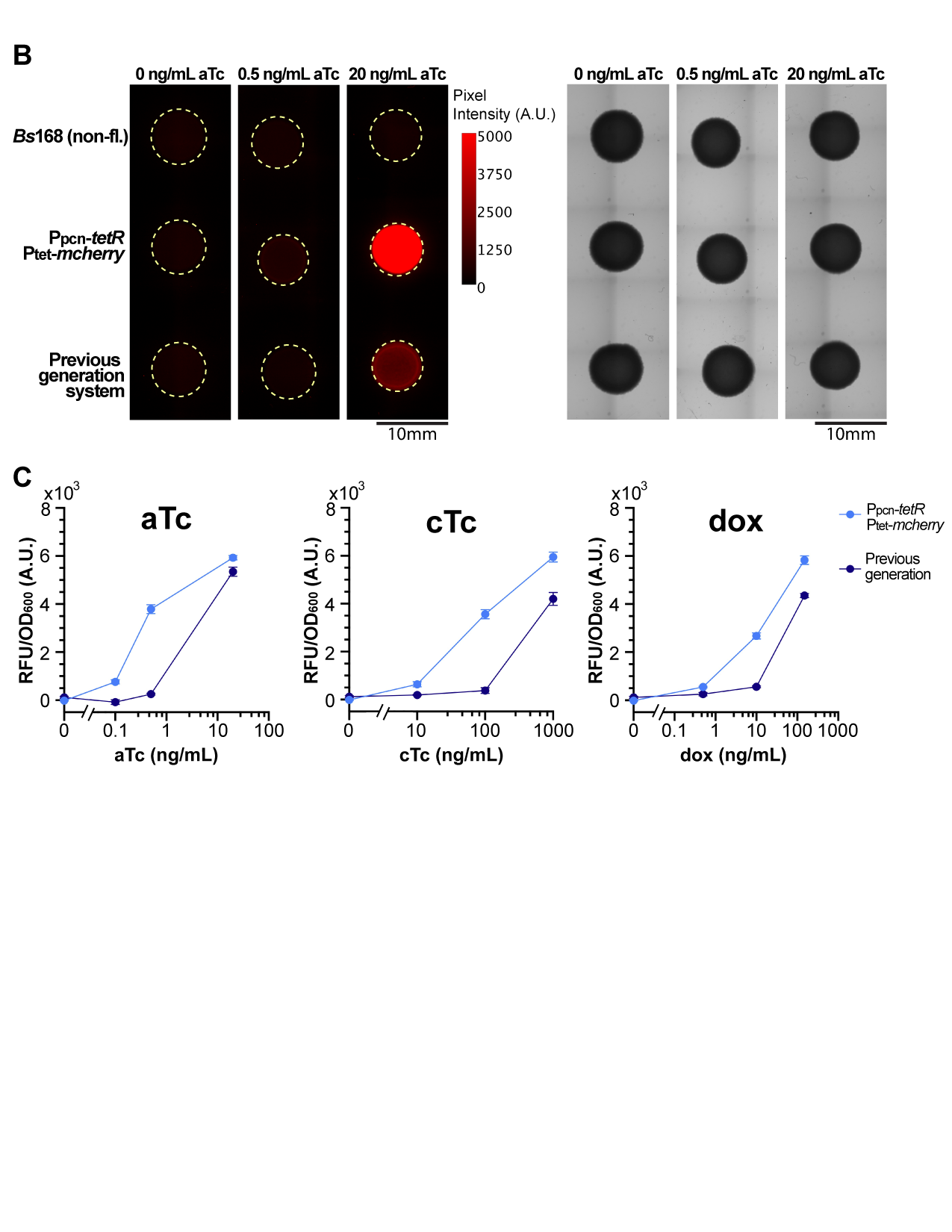
**

**Figure S3:** **Relative** **tetracycline induction of the improved system compared to a previous generation.**  (A) Relevant sequence comparisons of unoptimized system and current system. i) Protein sequence of TetR(B). DNA sequences of RBS region and *tetR* gene for ii) the previous generation system and iii) the ~codon-harmonized current system. Deviations from the original nucleotide sequence highlighted in blue. (B) Representative mCherry fluorescence (left) of colonies grown on minimal glucose agar plates supplemented with ~EC50 and maximal tested aTc concentration with corresponding brightfield images (right). Dashed circles indicate colony boundaries. (C) Dose-response induction using aTc, cTc and dox at ~mid-log (OD_600_ ~0.3) in minimal glucose media of improved system (light blue) and original system (dark blue). Dots and bars indicate the mean and SEM across three biologically independent experiments.

**
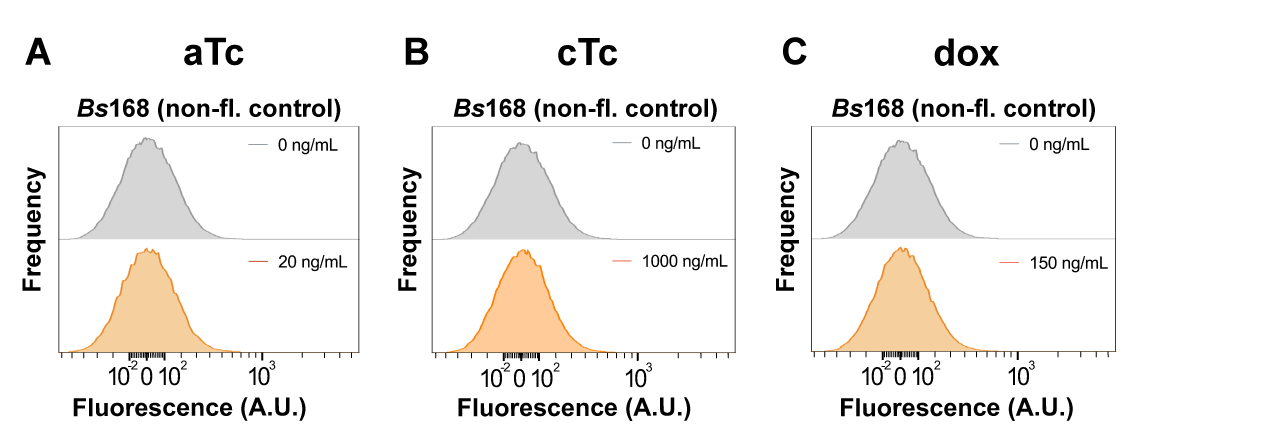
**

**Figure S4.** **Single cell distributions for nonfluorescent control treated with tetracycline in minimal glucose media.** Flow cytometry histograms measuring fluorescence of a *Bs*168 *trpC2* non-fluorescent control at 0 ng/mL and maximum tested concentration for (A) aTc, (B) cTc, and (C) dox in minimal glucose media from at least 40,000 events.

**
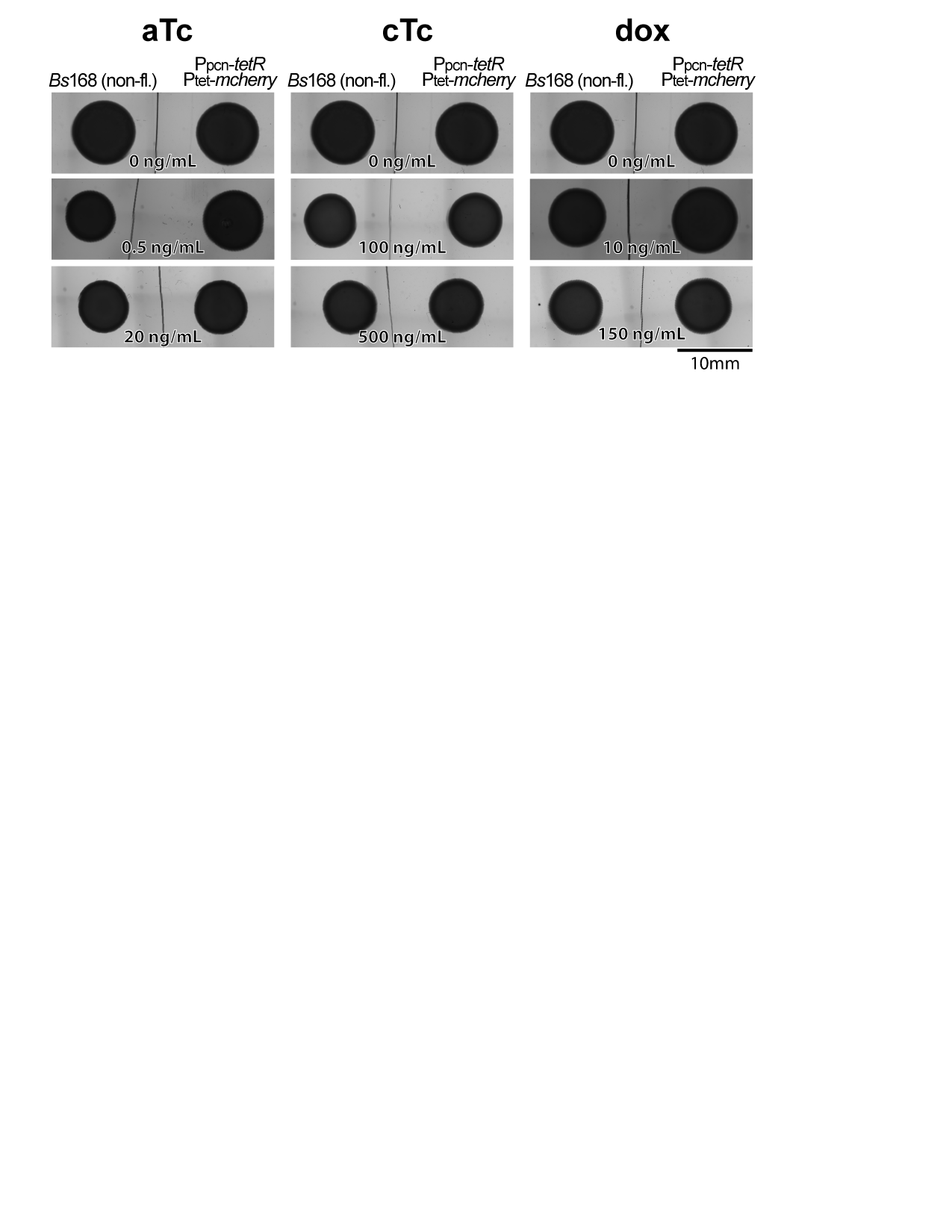
**

**Figure S5: Brightfield images of colonies of a nonfluorescent control and inducible strains grown on solid media.** Brightfield images of *B. subtilis* colonies on minimal glucose agar plates supplemented with tetracyclines corresponding to Figure 3.

**
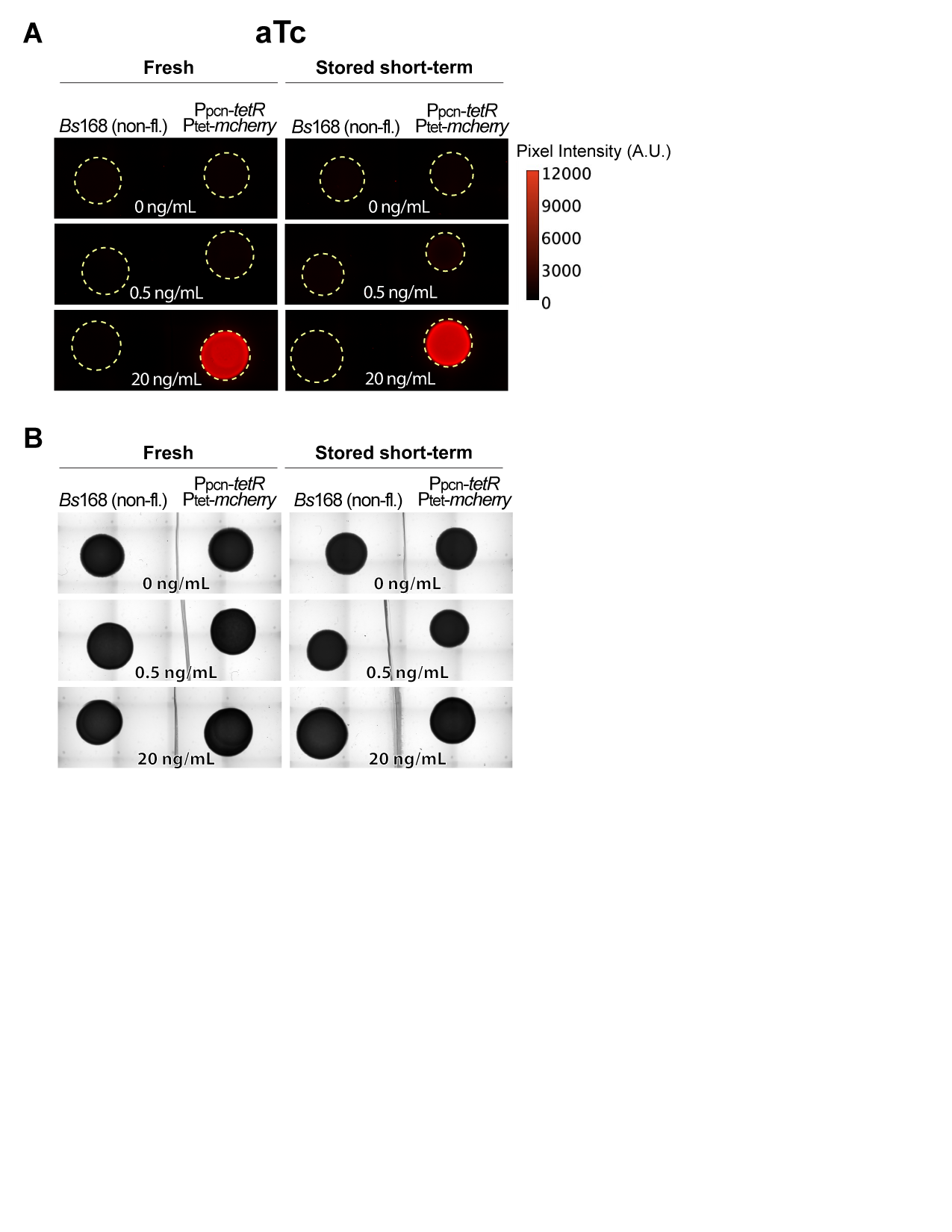
Figure S6:** **Anhydrotetracycline induction on fresh and short-term aged minimal glucose solid media.** (A) Representative mCherry fluorescence of colonies grown on minimal glucose agar plates supplemented with aTc on freshly poured plates (day 1, left) or short term aged plates (day 3, right), stored at 4°C sheltered from light prior to spotting. Fluorescence values were background- subtracted and normalized to the maximal induction condition for each day (Maximal values, Day 1: ~ 8571 ± 744; Day 3: ~ 9475 ± 469). At the liquid ~EC50 aTc concentration (0.5 ng/mL), normalized induction values on solid media were comparable between fresh and aged plates (Percent of max, Day 1: ~4.0% ± 0.9%; Day 3: ~4.7% ± 0.8%). Dashed circles indicate colony boundaries. (B) Corresponding brightfield images.

**
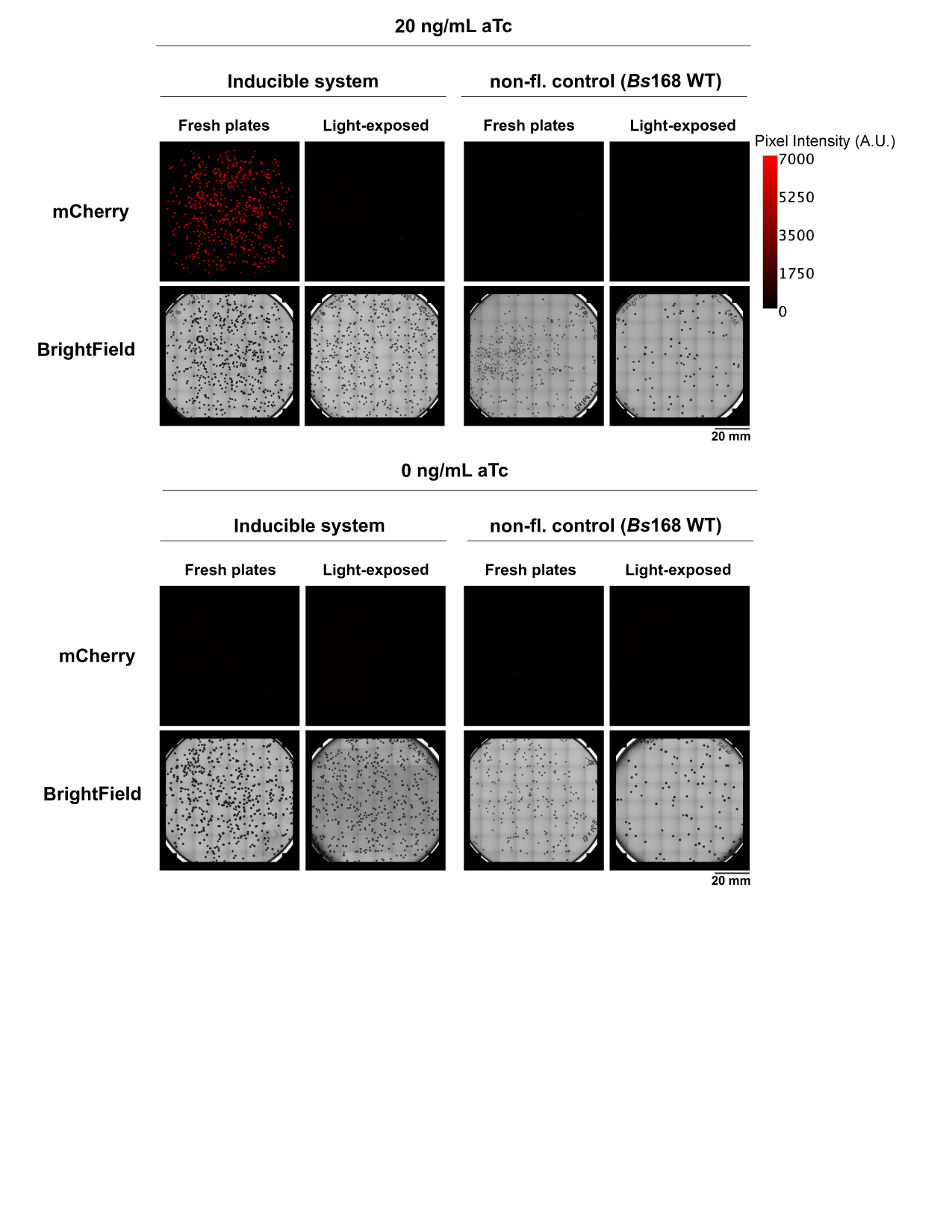
**

**Figure S7:** **Anhydrotetracycline induction on fresh and light-exposed minimal glucose solid media.** mCherry fluorescence of colonies grown on freshly prepared or light-exposed minimal glucose agar plates supplemented with aTc. Fresh plates were used on day 1 post-pouring. Light-exposed plates were kept at room temperature for ~10 days exposed to indoor ambient light. Maximal tested aTc concentration (top) is shown with no inducer (bottom) included for control. Corresponding brightfield images included.

**
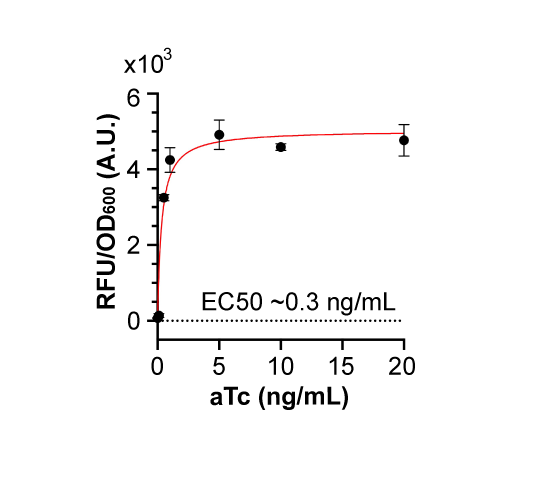
Figure S8:** **mCherry induction using pre-incubated and measurement cycled anhydrotetracycline under plate reader conditions in minimal glucose media.** Dose-response induction at ~mid-log (OD_600_ ~0.3) in minimal glucose media supplemented with aTc and subjected to excitation light cycles under plate reader conditions: 37°C, 560/20 nm, every 12 min for ~ 16 hr prior to inoculation. Red: Nonlinear regression fit with approximate EC50 value as indicated. Dots and bars indicate the mean and SD respectively of experimental triplicates.

**
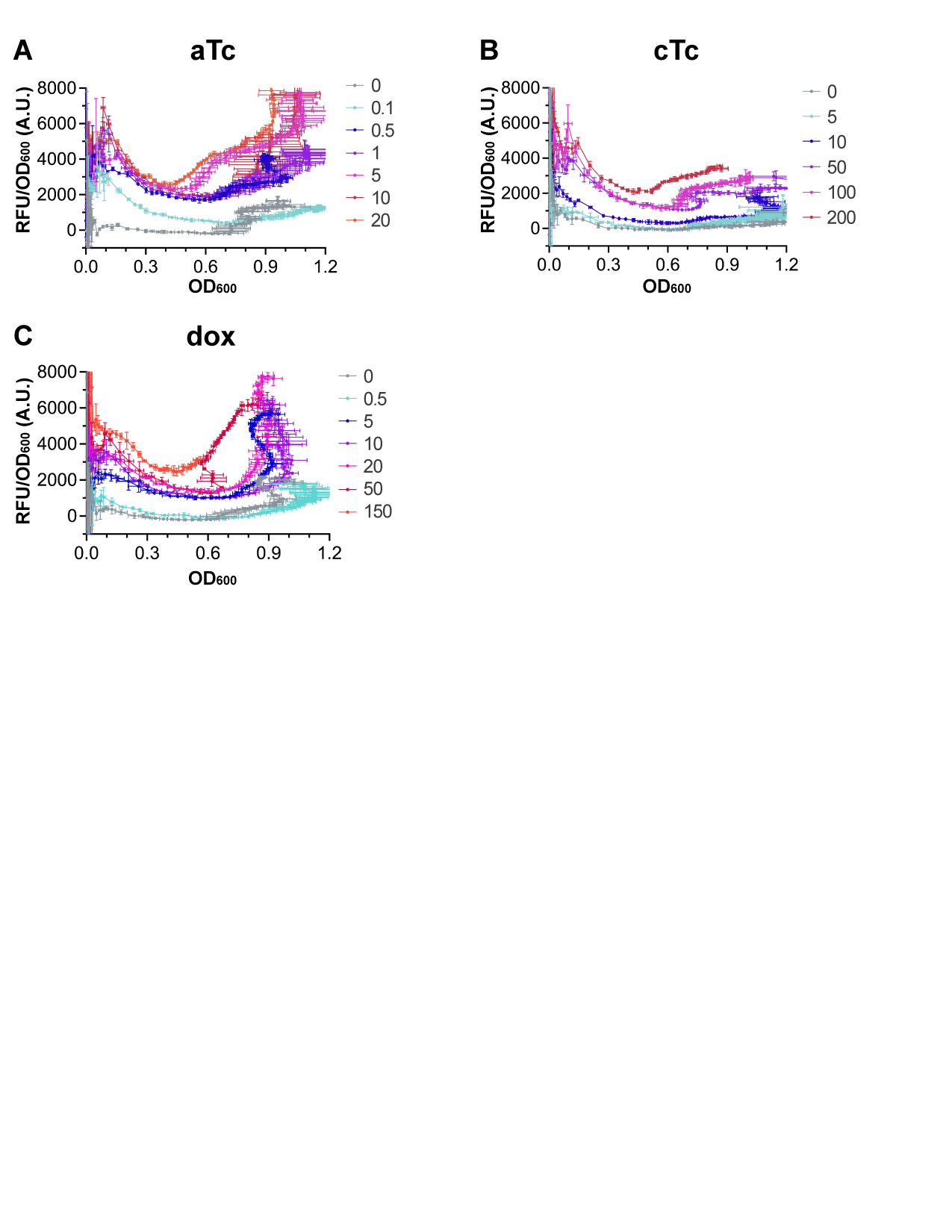
**

**Figure S9:** **mCherry fluorescence in rich media.** Representative average mCherry fluorescence for (A) aTc, (B) cTc and (C) dox (ng/mL) in CH media during bacterial growth. Error bars represent standard deviation across triplicates within a single experiment.


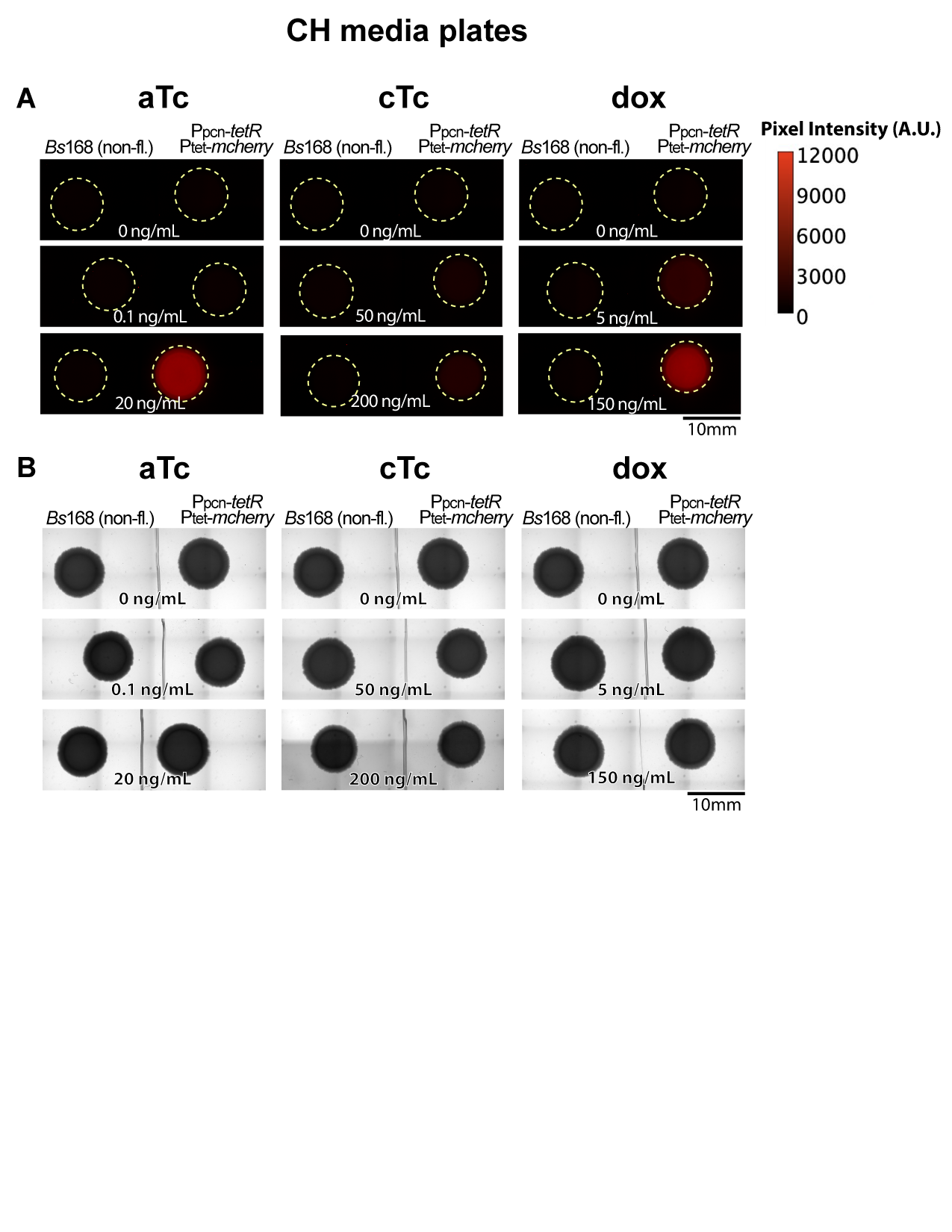


**Figure S10:** **Tetracycline induction on colonies grown on CH solid media.** (A) Representative mCherry fluorescence of colonies grown on CH agar plates supplemented with aTc, cTc and dox. Concentrations at each tetracycline’s ~EC50 in rich media and its maximal tested concentration are shown, with the 0 ng/mL condition shown for reference across all tetracyclines. Dashed circles indicate colony boundaries. (B) Corresponding brightfield images.

**
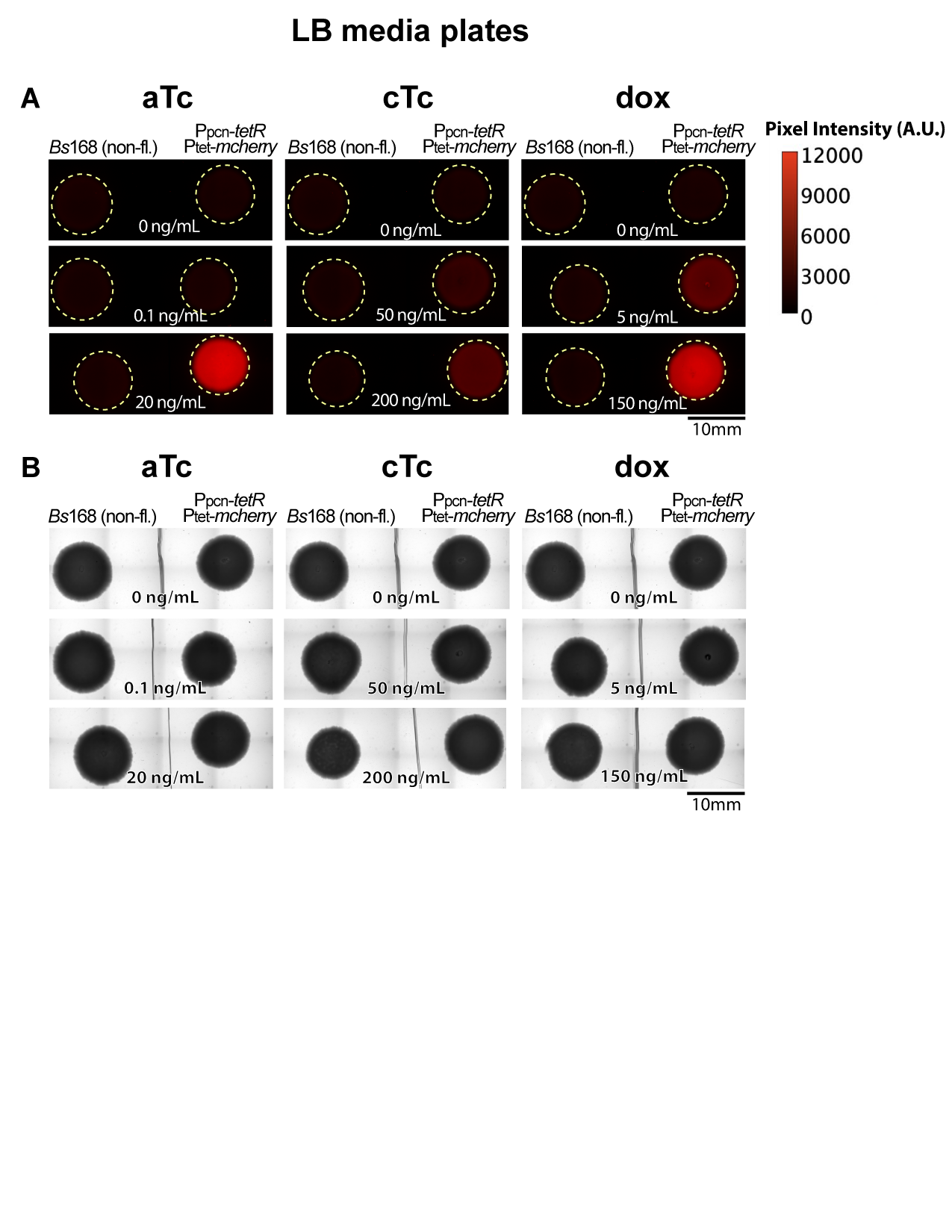
**

**Figure S11:** **Tetracycline induction on colonies grown on LB solid media.** (A) Representative mCherry fluorescence of colonies grown on LB agar plates supplemented with aTc, cTc and dox. Concentrations at each tetracycline’s ~EC50 in rich media and its maximal tested concentration are shown, with the 0 ng/mL condition shown for reference across all tetracyclines. Dashed circles indicate colony boundaries. (B) Corresponding brightfield images.


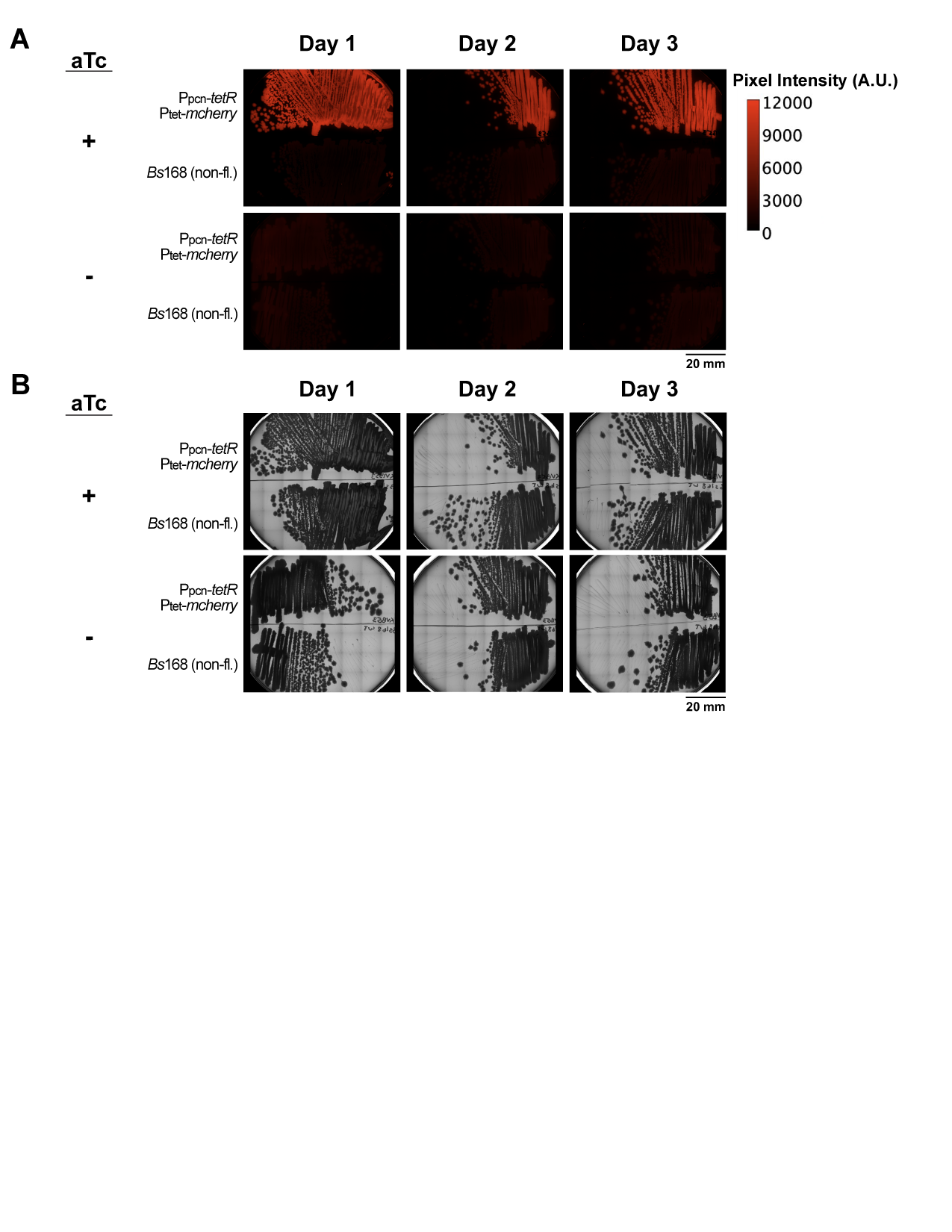


**Figure S12. Serial restreaking of chromosomally integrated system.** A single colony of the *amyE*-integrated construct serially restreaked for three consecutive days on LB plates ± 20 ng/mL aTc. (A) mCherry fluorescence and (B) corresponding brightfield plate images from each restreak.

**
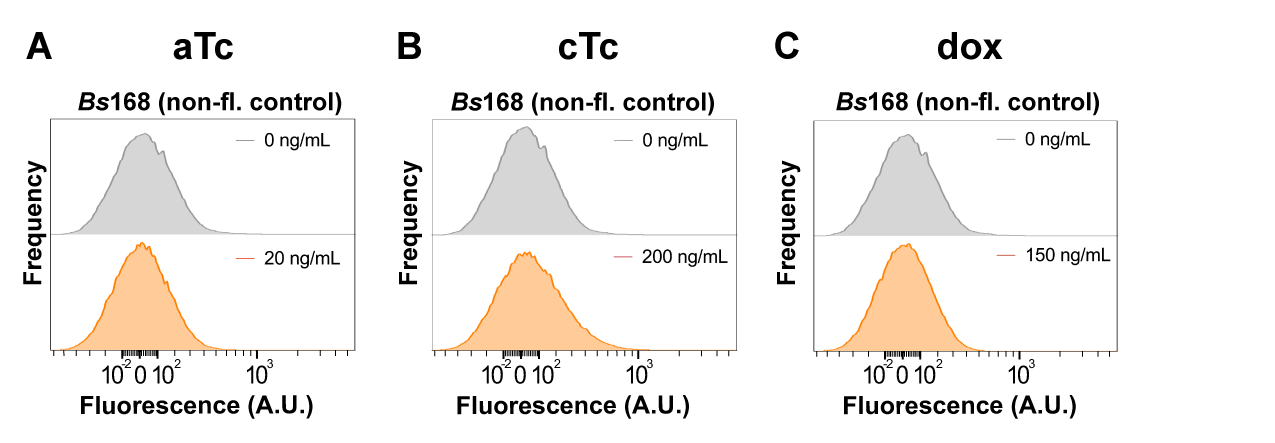
**

**Figure S13:** **Single cell distributions for nonfluorescent control treated with tetracycline in rich media.** Flow cytometry histograms measuring fluorescence of a *Bs*168 *trpC2* non-fluorescent control at 0 ng/mL and maximum tested concentration for (A) aTc, (B) cTc, and (C) dox in CH media from at least 40,000 events.

**
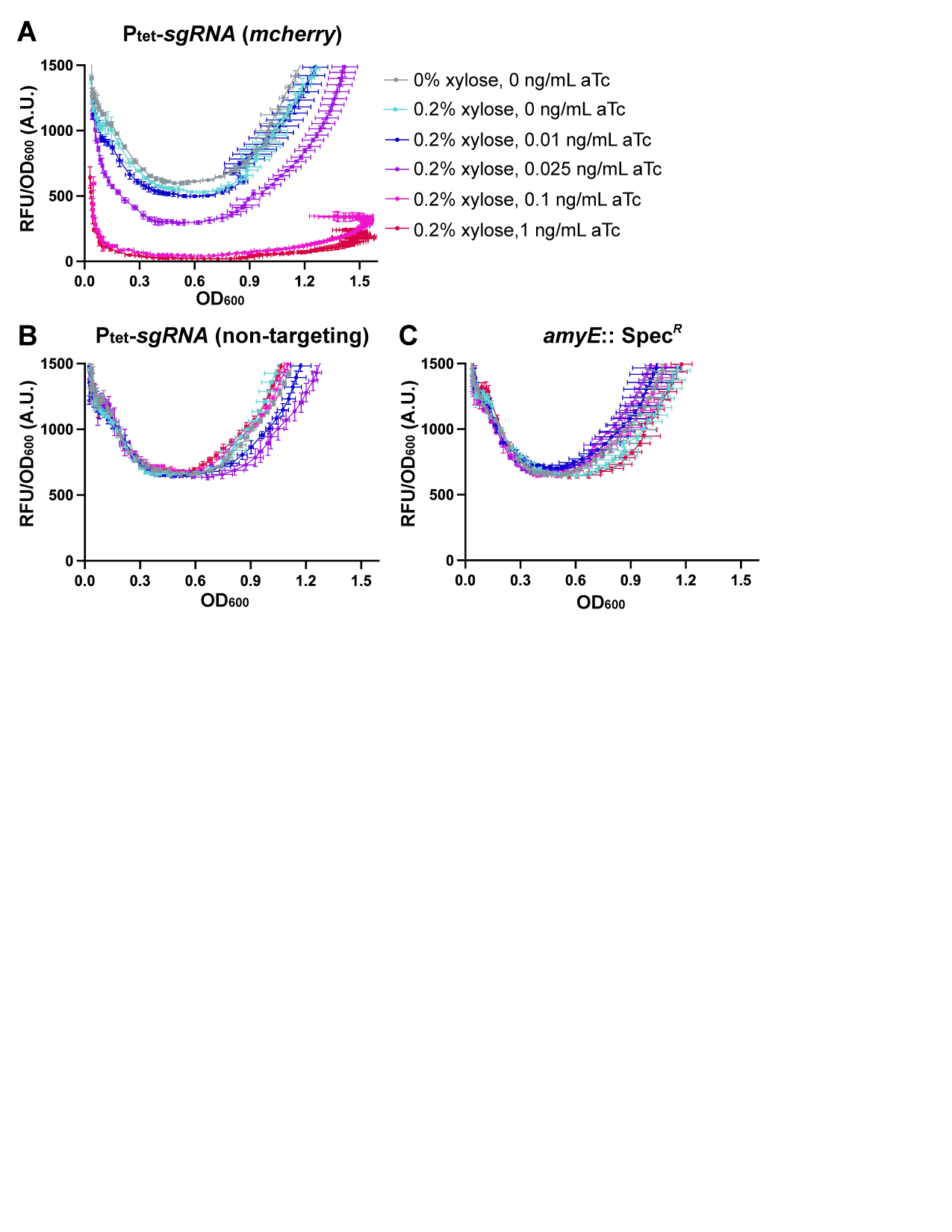
**

**Figure S14: mCherry repression using aTc-induced sgRNAs.** Representative mean mCherry fluorescence for (A) *mcherry*-targeting sgRNA, (B) non-targeting sgRNA and (C) an empty vector control (no sgRNA) in CH media during bacterial growth. Error bars represent standard deviation across triplicates within a single experiment.

**Supplementary Table S1.** List of strains used in this study

| **Strain** | **Genotype** | **Construction** | **Source** |
| --- | --- | --- | --- |
| *NEB 5α* | fhuA2Δ(argF-lacZ)U169 phoA glnV44 Φ80Δ(lacZ)M15 gyrA96 recA1 relA1 endA1 thi-1 hsdR17 |  | New England Biolabs (#C2987H) |
| *Bacillus subtilis* 168 *trpC2* |  |  | Lab stock |
| KVB53 | *amyE*:: P*_pcn_-tetR* P*_tet_*-*mcherry* Spec^R^ | Transformation of pKV67 into *Bs*168 *trpC2*, selecting on spectinomycin. | This work |
| KVB136 | *amyE*:: Spec^R^ | Transformation of pDG1730 into *Bs*168 *trpC2*, selecting on spectinomycin. | This work |
| KVB32 | *amyE*:: P*_pcn_-tetR* (not codon-harmonized or RBS improved) P*_tet_*-*mcherry* Spec^R^ | Transformation of pKV40 into *Bs*168 trpC2, selecting on spectinomycin | This work |
| AERB2 | *ganA:: xylR* P*_xyl_-dCas9* Erm^R^ | Transformation of pAER001 into *Bs168 trpC2*, selecting on MLS. | This work |
| AERB37 | *amyE::* P*_pcn_-tetR* P*_tet_-sgRNA (mcherry-targeting)* Spec^R^ | Transformation of pAER014 into *Bs*168 *trpC2*, selecting on spectinomycin | This work |
| AERB66 | *amyE::* P*_pcn_-tetR* P*_tet_-sgRNA (mcherry-targeting)* Spec^R^  *ganA:: xylR* P*_xyl_-dCas9* Erm^R^ | Transformation of AERB2 into AERB37, selecting on MLS. | This work |
| AERB74 | *amyE::* P*_pcn_-tetR* P*_tet_-sgRNA (non-targeting)* Spec^R^ | Transformation of pAER022 into *Bs168 trpC2*, selecting on spectinomycin | This work |
| AERB76 | *sacA::* P*_veg-_mcherry* Cm^R^ | Transformation of pAER021 into *Bs168 trpC2*, selecting on chloramphenicol | This work |
| AERB77 | *ganA:: xylR* P*_xyl_-dCas9* Erm^R^ *amyE::* P*_pcn_-tetR* P*_tet_-sgRNA (non-targeting)* Spec^R^ | Transformation of AERB74 into AERB2, selecting on spectinomycin | This work |
| AERB78 | *amyE::* Spec^R^  *ganA:: xylR* P*_xyl_-dCas9* Erm^R^ | Transformation of KVB136 into AERB2, selecting on spectinomycin | This work |
| AERB82 | *amyE::* P*_pcn_-tetR* P*_tet_-sgRNA (mcherry-targeting)* Spec^R^  *sacA::* P*_veg_ mcherry* Cm^R^  *ganA:: xylR* P*_xyl_-dCas9* Erm^R^ | Transformation of AERB76 into AERB66, selecting on chloramphenicol | This work |
| AERB83 | *amyE:: P_tet_-sgRNA (non-targeting)* Spec^R^  *sacA::* P*_veg_-mcherry* Cm^R^ *ganA:: xylR* P*_xyl_-dCas9* Erm^R^ | Transformation of AERB76 into AERB77, selecting on chloramphenicol | This work |
| AERB84 | *amyE::* Spec^R^  *sacA::* P*_veg_-mcherry* Cm^R^ *ganA:: xylR* P*_xyl_-dCas9* Erm^R^ | Transformation of AERB76 into AERB78, selecting on chloramphenicol | This work |

**Supplementary Table S2.** List of plasmids used in this study

| **Plasmid** | **Genotype** | **Construction** | **Source** |
| --- | --- | --- | --- |
| CZ82 | P_veg_-*mcherry* Amp^R^ Kan^R^ |  | Lab stock, *P_veg_-mcherry* sequence from (1) |
| pSac-Cm | Cm^R^ Amp^R^; Shuttle vector for integration at *sacA* |  | Lab Stock (2) |
| pDG1730 | Amp^R^ Spec^R^ Erm^R^ *amyE homology* |  | Lab stock |
| pKV40 | P*_pcn_*-*tetR* (not codon-harmonized or RBS improved) P*_tet_*-*mcherry* Amp^R^; Shuttle vector for integration at *amyE* | Backbone amplified from pDG1730 using KVp91 and KVp92. *P_pcn_-tetR P_tet_-mcherry* vector amplified from previous generation lab stock plasmid using primers KVp93 and KVp94. Assembled through Golden Gate cloning using primer-introduced PaqCI sites. | This work |
| pKV103 | *RBS tetR* P*_AmpR_*-Amp^R^ lacZα | The DNA sequence for a codon-harmonized *tetR* gene with a RBS was ordered for gene synthesis through Azenta cloned into their standard pUC-GW-Amp plasmid backbone. | This work |
| pKV67 | P*_pcn_*-*tetR* P*_tet_*-*mcherry* Amp^R^; Shuttle vector for integration at *amyE* | Backbone amplified from pKV40 using primers KVp150 and KVp151. Codon-harmonized *tetR* and RBS amplified from pKV103 plasmid using primers KVp152 and KVp153. Assembled through Golden Gate cloning using primer-introduced PaqCI sites. | This work |
| pAER001 | *xylR* P*_xyl_-dCas9* Erm^R^ Amp^R^ | Vector and dCas9 sequence from pJMP1(3) amplified with AERp007 and AERp010. Assembled through Golden Gate cloning using primer-introduced BsaI sites. | This work |
| pAER014 | P*_pcn_*-*tetR* P*_tet_*-*sgRNA (mcherry targeting)* Amp^R^ | Vector amplified from pKV67 using AERp097 and AERp098. sgRNA insert (sequence listed below) and downstream *Spec^R^* amplified from pJMP3-derived plasmid (3) using AERp100 and AERp101. Assembled through Golden Gate cloning using primer-introduced BsaI sites. | This work |
| pAER021 | P*_veg_-mCherry*  Amp^R^ | P*_veg_-mCherry* sequence from CZ82 cloned into pSac-Cm. Assembled via site-directed mutagenesis. | This work |
| pAER022 | P*_pcn_*-*tetR* P*_tet_*-*sgRNA (non-targeting)* Amp^R^ | pAER014 was amplified with AERp121 and AERp122, introducing a non-targeting sgRNA with primer overhangs. Assembled via site-directed mutagenesis. | This work |

**Supplementary Table S3**. List of oligos used in this study

| **Name** | **Sequence (5'-3')** |
| --- | --- |
| KVp91 | ggctaccacctgccatgacaaataaCGAATTCCTGCAGCCCTG |
| KVp92 | ggctaccacctgccaaatgggtcttgaATAAGCTTCTAGGATCCGATCAG |
| KVp93 | ggctaccacctgccaaaCCCAGATTCGCATTTTAAC |
| KVp94 | ggctaccacctgccatgTTGTACAGCTCATCCATG |
| KVp150 | ggctaccacctgcatgtatcaTTGATCGTAACCAGATGAAG |
| KVp151 | ggctaccacctgccaagAGGATCCGATCAGACCAG |
| KVp152 | ggctaccacctgccaagtcctagaagcttatTTATGAGCCGCTCTCACATTTG |
| KVp153 | ggctaccacctgcatgttgatCGATGAACAGGAGGTTC |
| AERp007 | ggctacggtctcatgACGTATGGATAAGAAATACTCAATAGGC |
| AERp010 | ggctacggtctccgtcacctccTTTTAGATATCACTAGTTTGGACC |
| AERp097 | ggctaccacctgctggcATTATAAAAAAATTGAAAAAATGGTGGAAAC |
| AERp098 | ggctaccacctgcccgaTTGTCCTCCTTATTAGTTCTC |
| AERp100 | ggctaccacctgctggcTAATTTTTTTAATCTGTTATTTAAATAGTTTATAG |
| AERp101 | ggctaccacctgcccgaacaaacGATAGTCCGGGATATCCGCT |
| AERp119 | TCCTAAATTCACTTTAGATAAAAATTTAGGAGGC |
| AERp122 | GTTTGTCCTCCTTATTAGTTCTC |

CAPS: homology to template

**Supplementary Table S4.** DNA and protein sequences used in this study

| **Name** | **DNA Sequence** |
| --- | --- |
| P*_pcn_* promoter (4), the -35 and -10 (underlined) | cggtggaaacgaggtcatcatttccttccgaaaaaacggttgcatttaaatcttacatatgtaatactttcaaagactacatttgtaagatttg |
| Ribosome Binding Site (RBS) region before *tetR* in KVB53 | aggaggttcata |
| Codon-harmonized *tetR* gene for *B. subtilis* in KVB53 | ATGTCCAGACTTGATAAGAGTAAAGTAATCAACTCTGCGCTGGAATTGTTGAATGAAGTTGGTATTGAGGGCTTAACCACGCGGAAGTTGGCACAGAAACTCGGGGTCGAGCAGCCTACGCTGTACTGGCACGTCAAGAACAAAAGAGCTTTGCTCGATGCCCTCGCCATAGAGATGTTGGATCGGCATCATACGCATTTTTGCCCGCTCGAAGGTGAGAGCTGGCAGGATTTTCTCCGGAACAATGCTAAATCATTTCGCTGCGCACTCTTATCTCATCGTGATGGCGCGAAGGTTCACCTTGGAACACGCCCTACTGAAAAACAGTATGAAACGCTGGAGAACCAATTAGCGTTTCTTTGCCAACAAGGATTTTCTTTGGAAAACGCCTTATACGCGCTCTCTGCTGTTGGACACTTTACACTCGGATGTGTGCTGGAGGATCAGGAACATCAGGTCGCCAAGGAAGAGCGTGAAACTCCGACAACTGATTCTATGCCGCCGTTACTTCGGCAGGCCATAGAACTTTTCGATCACCAAGGCGCGGAACCGGCTTTCCTTTTCGGCCTTGAGTTAATTATCTGTGGCCTGGAAAAGCAACTCAAATGTGAGAGCGGCTCATAA |
| TetR (class B) protein sequence | MSRLDKSKVINSALELLNEVGIEGLTTRKLAQKLGVEQPTLYWHVKNKRALLDALAIEMLDRHHTHFCPLEGESWQDFLRNNAKSFRCALLSHRDGAKVHLGTRPTEKQYETLENQLAFLCQQGFSLENALYALSAVGHFTLGCVLEDQEHQVAKEERETPTTDSMPPLLRQAIELFDHQGAEPAFLFGLELIICGLEKQLKCESGS |
| P*_tet_* promoter with ***tetO_1_***, ***tetO_2_*** (bold). The -35 and -10 for sigma A (underlined) (5) | aattttgtcaaaataattttattgacaacgtcttattaacgttgataccggttaaattttatttgac**actctatcattgatagagt**acaataaatac**tccctatcagtgatagaga** |
| Ribosome Binding Site (RBS) region before *tetR* in KVB32 | agggagacgattttg |
| DNA sequence of *tetR* used in previous generation system in KVB32 | ATGTCCAGATTGGATAAGAGTAAGGTGATCAACTCAGCATTGGAGCTGTTGAATGAAGTGGGCATAGAGGGACTCACCACTCGCAAGCTGGCACAAAAACTGGGGGTTGAGCAGCCAACCCTTTACTGGCACGTCAAGAATAAGCGTGCGCTCTTGGATGCACTTGCCATAGAGATGTTAGACCGTCACCATACGCATTTCTGTCCATTGGAAGGCGAGTCTTGGCAAGATTTTCTGAGAAATAATGCTAAGAGCTTTAGATGCGCCCTCCTGTCACACCGCGACGGGGCCAAAGTCCACCTGGGTACCAGACCGACCGAAAAGCAGTACGAGACTTTAGAGAATCAGCTCGCATTTCTCTGCCAGCAAGGGTTTTCATTAGAAAACGCGCTTTACGCCCTGTCAGCTGTGGGTCACTTCACGTTAGGATGTGTCCTTGAAGATCAAGAACACCAGGTTGCGAAGGAAGAACGGGAGACTCCGACAACTGATTCTATGCCACCTTTGTTACGGCAAGCTATCGAGCTCTTTGATCACCAAGGTGCGGAACCTGCTTTCTTGTTTGGTTTGGAACTCATTATATGCGGCTTGGAGAAACAGTTAAAATGCGAATCTGGGTCTTGA |
| sgRNA targeting *mcherry* spacer sequence | gatagtccgggatatccgct |
| Non-targeting sgRNA spacer sequence | cagtaaggagagcttcaact |

**References**

1. Libby EA, Reuveni S, Dworkin J. 2019. Multisite phosphorylation drives phenotypic variation in (p)ppGpp synthetase-dependent antibiotic tolerance. Nature Communications 10:5133. <https://doi.org/10.1038/s41467-019-13127-z>

2. Middleton R, Hofmeister A. 2004. New shuttle vectors for ectopic insertion of genes into *Bacillus subtilis*. Plasmid 51:238-245. <https://doi.org/10.1016/j.plasmid.2004.01.006>

3. Peters JM, Colavin A, Shi H, Czarny TL, Larson MH, Wong S, Hawkins JS, Lu CHS, Koo B-M, Marta E, Shiver AL, Whitehead EH, Weissman JS, Brown ED, Qi LS, Huang KC, Gross CA. 2016. A Comprehensive, CRISPR-based Functional Analysis of Essential Genes in Bacteria. Cell 165:1493-1506. <https://doi.org/10.1016/j.cell.2016.05.003>

4. Castillo-Hair SM, Fujita M, Igoshin OA, Tabor JJ. 2019. An Engineered B. subtilis Inducible Promoter System with over 10 000-Fold Dynamic Range. ACS Synthetic Biology 8:1673-1678. <https://doi.org/10.1021/acssynbio.8b00469>

5. Annette K, Ralph B, Wolfgang H. 2005. Tetracycline-Dependent Conditional Gene Knockout in Bacillus subtilis. Applied and Environmental Microbiology 71:728-733. <https://doi.org/10.1128/AEM.71.2.728-733.2005>
